# A Special Request

**Published:** 1887-02

**Authors:** 


### A Special Request.

The Chairman of the Board of Dental Examiners of the State of Kentucky desires to have at the earliest moment a complete registration of all the dentists practicing within that State, and it is desirable that the names of all such be sent to him at once, and parties in the various points in the State who may be registered are requested to give the names of any whom they may know as practitioners in their respective districts of country whose names may not have heretofore been given to the board for registration. It is earnestly desired by the board that special attention of the dentists throughout the State be given to this matter, especially giving the names of any who may be practicing in violation of the law. All such names and information should be sent to Dr. A. O. Rawls, Lexington, Ky.

